# Off-training physical activity and training responses as determinants of sleep quality in young soccer players

**DOI:** 10.1038/s41598-021-89693-4

**Published:** 2021-05-13

**Authors:** Nuno Mateus, Juliana Exel, Bruno Gonçalves, Anthony Weldon, Jaime Sampaio

**Affiliations:** 1 Research Center in Sports Sciences, Health Sciences and Human Development, CIDESD, CreativeLab Research Community, Vila Real, Portugal; 2 Department of Sports Science, Exercise and Health, School of Life Sciences and Environment, University of Tras-os-Montes and Alto Douro, Vila Real, Portugal; 3 Centre for Sport Science and University Sports, Department of Biomechanics, Kinesiology and Computer Science in Sport, University of Vienna, Vienna, Austria; 4 Departamento de Desporto e Saúde, Escola de Saúde e Desenvolvimento Humano, Universidade de Évora, Évora, Portugal; 5 Comprehensive Health Research Centre (CHRC), Universidade de Évora, Évora, Portugal; 6 Portugal Football School, Portuguese Football Federation, Oeiras, Portugal; 7 Technological and Higher, Education Institute of Hong Kong (THEi), Hong Kong, China

**Keywords:** Health care, Health occupations, Medical research

## Abstract

This study aimed to quantify and assess the relationship of young soccer players' off-training physical activity (PA) and training responses on sleep quality. Eleven adolescent soccer players (13 ± 0.5 years old) were monitored during weekdays for four consecutive weeks, throughout soccer practice days. Off-training PA and sleep quality were assessed using 100 Hz tri-axial accelerometers and training responses analyzed using 20 Hz global positioning measurement units. A cluster analysis classified all cases into three different dimensions, (1) off-training PA, (2) training responses and (3) sleep quality. For each dimension, the most important variables for classifying the cases into clusters were sedentary PA and moderate-to-vigorous PA; total distance covered and impacts; and sleep onset latency and sleep fragmentation index, respectively. Afterwards, a correspondence analysis was used to identify whether off-training PA and training responses affected sleep quality. Results exposed that high to medium off-training PA combined with medium to high training responses may have decreased sleep quality. Conversely, no correspondence was observed between off-training PA and training responses, with higher sleep quality. This study emphasizes the importance of sports organizations adopting a holistic approach to youth soccer players’ development, that appropriately considers the inter-relationship between lifestyle, performance and health-related information.

## Introduction

The role of lifestyles, physical activity (PA) and sedentary behaviors (SB) on health-related aspects have been well established within general populations^1,2^. This has led to greater interest in the daily PA and SB of athletes. Although a recent systematic review^3^, established athletes achieve the weekly recommended moderate-to-vigorous physical activity (MVPA) guidelines, it has also been observed athletes have excessive SB during off-training time^4,5^. It is often misconceived that young athletes are active and immune to this issue, however literature illustrates they also exhibit high SB^6^. This is further supported through research identifying similar rates of SB between athletes and non-athletes in various sports and age groups^6–8^. Whereas, family and health organization members may have also overlooked the negative effects of SB, while perceiving sports training to be sufficient in overcoming associated issues. However, recent evidence suggests that SB is independent from PA and may be associated with decreased weight control, academic performance impairment, risk of insomnia and sleep disturbance^9–11^. While a positive association between children and adolescents PA levels and muscular fitness has been observed^12^. Such findings highlight the importance of quantifying and understanding the impact of young athletes’ lifestyles outside of organized sports training to make robust inferences for health and athletic performance.

Regarding sports performance, off-training physical behavior may impact the balance of training loads for performance optimization. To date, only one study has investigated the relationship between off-training PA and training performance, concluding that PA preceding soccer training is positively correlated with PA intensity during practice^13^. Furthermore, youth soccer training is becoming increasingly demanding and structured, with coaches frequently prescribing training demands similar to match intensity, to optimize players' high-performance levels^14–16^. However, youth players are still reaching maturation (e.g., body size and physiological function), and spikes in training workload commonly observed in team sports such as youth soccer is not the most suitable training approach^17^. With such demanding training loads, this may lead to excessive daily energy requirements of young players^17^. In particular, young talented players should be careful when moving into systematic or accelerated development training programs^18^, as frequently challenging the boundaries of players, may increase the likelihood of wellness issues.

To recover from high training demands a comprehensive recovery strategy should also be adopted^19^. For example in youth soccer players, sleep is considered fundamental for physiological and psychological recovery^20,21^. Furthermore, adequate sleep duration and quality are positively associated with health, cognitive function, and academic grades^22,23^. Whereas, sleep deprivation negatively influences physiological functions (e.g., immune function), psychological state (e.g., mood), academic performance (e.g., cognitive disturbance, attention and concentration) and sports performance (e.g., skill execution, recovery, injury risk)^20,22,24,25^. Research also suggests that young soccer players have a higher propensity towards later bedtimes and subsequent wake times compared to non-athletes^23^. Besides sports training, young athletes are also required to attend school and meet family and social obligations, which may contribute towards sleep disruption^20,26^. Therefore, it may be inferred that young players are at an increased risk of experiencing reduced sleep quality.

Overall, youth sports organizations are familiarized with wellbeing concepts for young players^27^. However, insufficient evidence is available to clarify how players lifestyles and competitive sports commitments influence wellness determinants, such as sleep quality. Therefore, it is important to further investigate the relationship between factors influencing youth players development such as off-training behavior, sports training workload and sleep. The purpose of this study was to quantify and assess the relationship of young soccer players' off-training PA and training responses on sleep quality.

## Results

**Figure 1:**
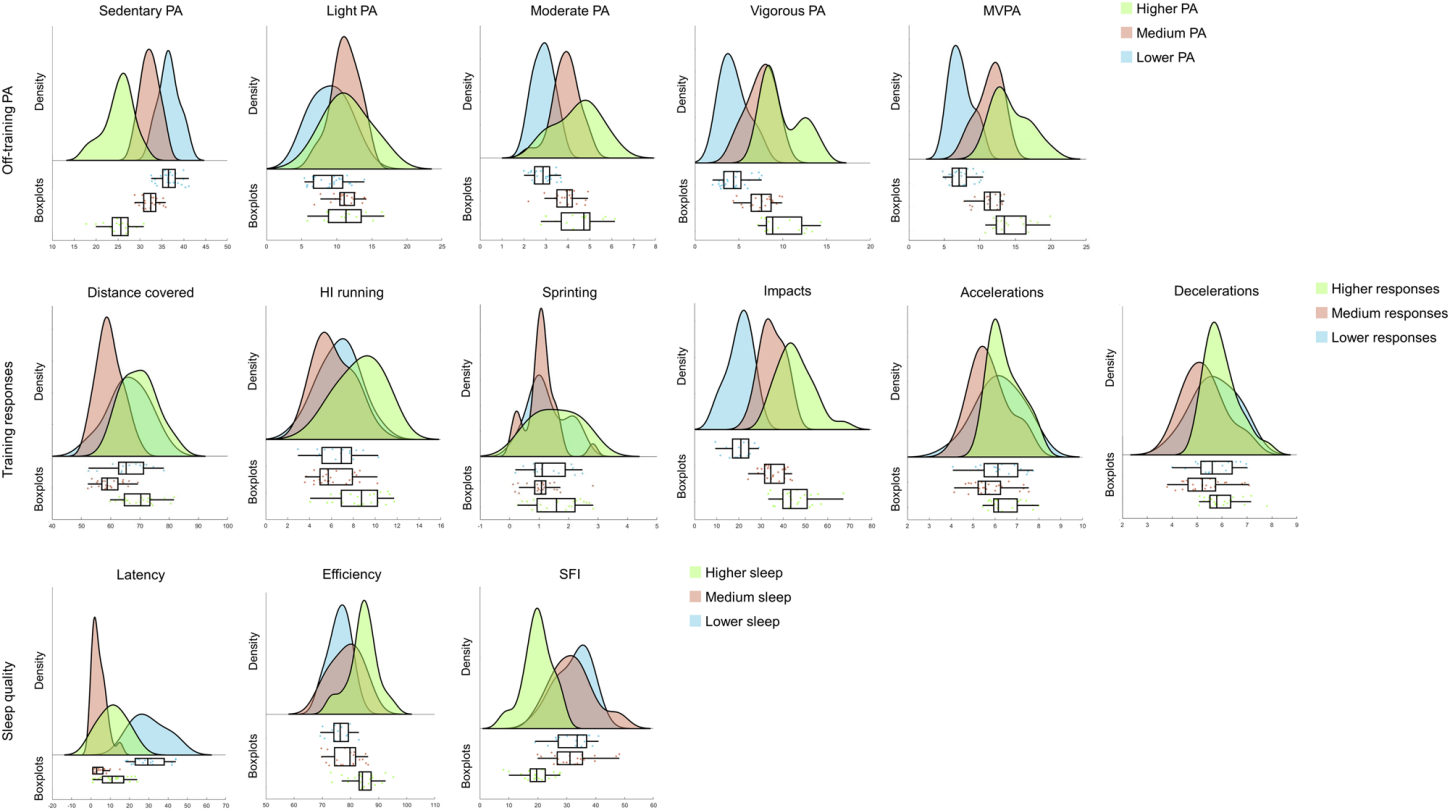
Raincloud plots showing the distribution of the variables according to the clusters obtained. The density plots indicate the data distribution and the boxplots indicate the data distribution, the median and the 1st quartile (25th percentile) and the 3rd quartile (75th percentile), for each group. *MVPA* moderate-to-vigorous physical activity, *PA* physical activity, *SFI* sleep fragmentation index.

The means and standard deviations of all variables according to each dimension cluster and the magnitude of F-values and Tukey HSD test are presented in Table 1. To complement, Fig. 1 presents the distribution of variables among the clusters obtained. Regarding off-training PA, variables such as sedentary PA, MVPA and vigorous PA were the principal criteria to discriminate clusters; conversely, light PA revealed less influence in differentiating clusters. Most data were grouped in cluster 1 (n = 28 data points), labeled as Lower PA, followed by cluster 2 (n = 22 data points), labeled as Medium PA and cluster 3 (n = 16 data points), labeled as Higher PA. Cluster Lower PA presented higher sedentariness (36.62 ± 2.31) and the lowest off-training activity in the remaining PA levels. Cluster Medium PA exposed a slightly lower amount of sedentary PA than the previous cluster, but significantly higher activity in the additional off-training PA levels. Cluster Higher PA revealed the lowest sedentariness (25.19 ± 3.36) and the higher activity in the other PA levels [e.g., vigorous PA (9.93 ± 2.29) and MVPA (14.39 ± 2.62)], among all off-training clusters.

**Table 1:** Means, standard deviations and predictor importance from the obtained clusters.

Variables/cluster		Lower PA (n = 28 data points)	Medium PA (n = 22 data points)	Higher PA (n = 16 data points)	F-values	Post-Hoc
Off-training PA	Sedentary PA	36.62 ± 2.31	32.27 ± 2	25.19 ± 3.36	105.51**	lm, lh, mh
	Light PA	9.01 ± 2.44	11.21 ± 1.94	11.45 ± 3.06	7.09**	lh
	Moderate PA	2.88 ± 0.46	3.89 ± 0.62	4.46 ± 1.01	30.54**	lm, lh
	Vigorous PA	4.43 ± 1.53	7.47 ± 1.57	9.93 ± 2.29	52.62**	lm, lh, mh
	MVPA	7.31 ± 1.56	11.36 ± 1.70	14.39 ± 2.62	74.19**	lm, lh, mh

Variables/cluster		Lower responses (n = 16 data points)	Medium responses (n = 26 data points)	Higher responses (n = 24 data points)	F-values	Post-Hoc
Training responses	Distance covered	66.14 ± 6.84	59.65 ± 4.26	69.65 ± 5.88	20.72**	mh, lm
	High-intensity running	6.67 ± 1.85	6.22 ± 1.73	8.58 ± 2.05	10.59**	mh, lh
	Sprinting	1.29 ± 0.66	1.07 ± 0.55	1.57 ± 0.78	3.53*	
	Impacts	20.42 ± 5.49	35.11 ± 5.24	45.36 ± 8.07	71.29**	lm, lh, mh
	Accelerations	6.21 ± 1	5.75 ± 0.88	6.47 ± 0.73	4.45*	mh
	Decelerations	5.72 ± 0.87	5.29 ± 0.85	5.98 ± 0.68	4.76*	mh

Variables/cluster		Lower sleep (n = 18 data points)	Medium sleep (n = 21 data points)	Higher sleep (n = 27 data points)	F-values	Post-Hoc
Sleep quality	Latency	30.17 ± 8.69	4.29 ± 3.65	11.37 ± 6.93	77.19**	lm, lh
	Efficiency	76.12 ± 3.71	78.39 ± 5.28	84.41 ± 5.09	18.20**	lh, mh
	SFI	32.18 ± 6.02	32.05 ± 7.44	19.98 ± 4.86	31.66**	lh, mh

*Lm* statistically significant differences between lower and medium groups, *lh* statistically significant differences between lower and higher groups, *mh* statistically significant differences between medium and higher groups, *PA* physical activity.

*Statistically significant differences at p < 0.05; **Statistically significant differences at p < 0.001.

Considering the training responses, the most critical variable for differentiating clusters was impacts; with accelerations, decelerations and sprinting showing less importance. Cluster 1 was labeled as Lower responses (n = 16 data points), cluster 2 was labeled as Medium responses (n = 26 data points), and cluster 3 was labeled as Higher responses (n = 24 data points). Cluster Lower responses demonstrated greater total distance covered (66.14 ± 6.84); however, revealed the lowest proportion of impacts (20.42 ± 5.49). Cluster Medium responses presented a slightly lower training workload than the previous cluster, but a notably higher number of impacts (35.11 ± 5.24). Cluster Higher responses displayed the highest mean for all training-related variables.

Sleep onset latency revealed a higher magnitude to differentiating the clusters; with sleep efficiency being less influential. Cluster 1 was labeled as Lower sleep (n = 18 data points), cluster 2 was labeled as Medium sleep (n = 21 data points), and cluster 3 was labeled as Higher sleep (n = 27 data points). Cluster Lower sleep disclosed the higher latency (30.17 ± 8.69) and sleep fragmentation index (SFI) (32.18 ± 6.02), but the lowest efficiency (76.12 ± 3.71). Cluster Medium revealed slightly lower SFI (32.05 ± 7.44) and higher efficiency (78.39 ± 5.28) compared to the aforementioned cluster, and the lowest latency. Finally, cluster Higher sleep showed the highest efficiency (84.41 ± 5.09) and the least SFI (19.98 ± 4.86) among sleep clusters.

**Figure 2:**
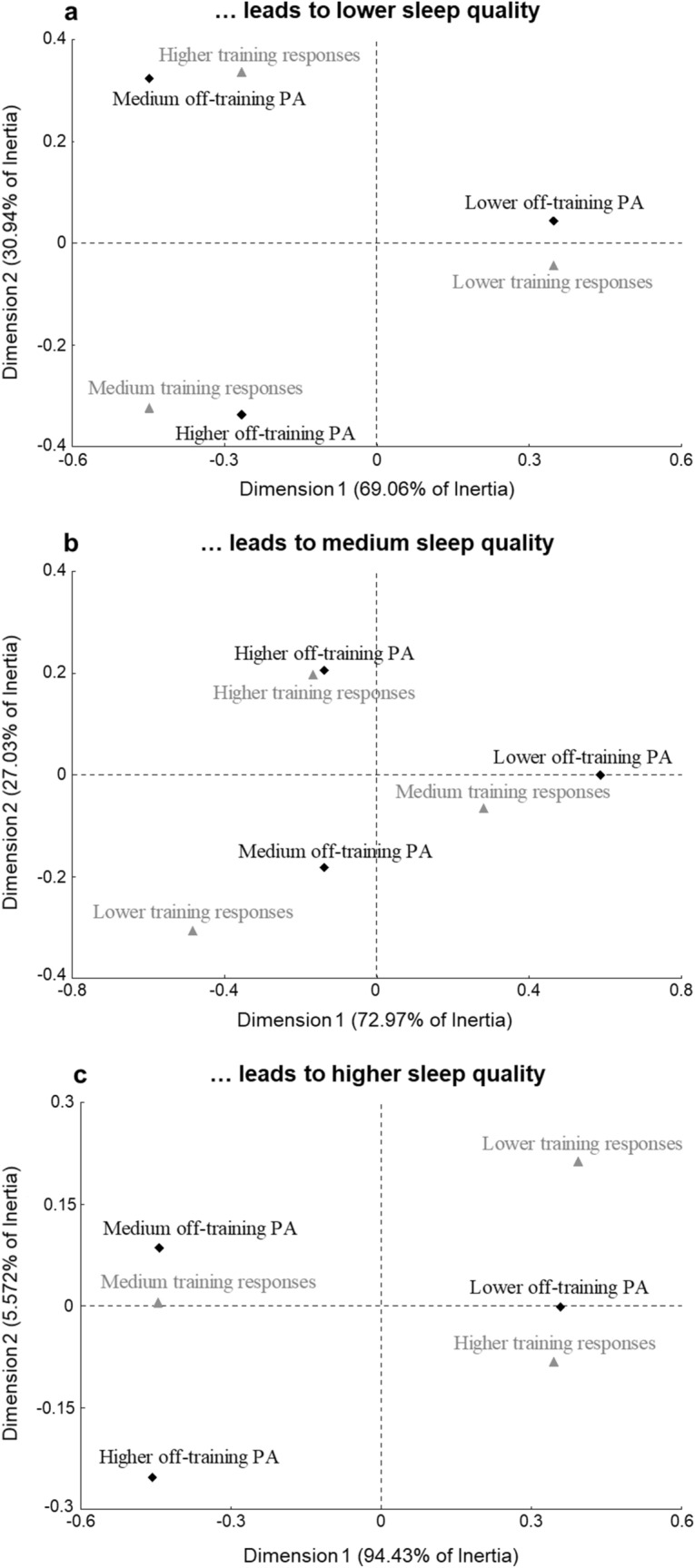
(**a**–**c**) Bi-plot from the correspondence analysis of off-training PA and training responses, according to sleep quality. (**a**) Leads to lower sleep quality; (**b**) leads to medium sleep quality; (**c**) leads to higher sleep quality. *PA* physical activity.

The process of summarizing the interactions between rows (off-training PA) and columns (training responses) was conducted for all sleep groups, whereby the outputs are presented as a solution of two dimensions. For the Lower sleep group results showed the first dimension was characterized by Lower off-training PA (as the relative inertia and cosine^2^ values exhibit) (see Table 2 and Fig. 2a) and a second dimension was most related to the Higher off-training PA profile. In the same sleep group, a similar trend was observed for Lower training responses (dimension 1, 69.06% of inertia) in contrast with the Higher training responses (dimension 2, 30.94% of inertia). The quadrants in Fig. 2a, suggest that Higher training responses are near the Medium off-training PA, that Medium responses are near the Higher off-training PA and there is independence from Lower off-training PA and Lower training response (these categories are located separately in different quadrants).

**Table 2:** Description of the results obtained in the correspondence analysis for sleeping patterns of the three groups.

Variables/dimension		Dimension 1		Dimension 2	
		Inertia	Cosine^2^	Inertia	Cosine^2^
Lower sleep	Lower off-training PA	0.48	0.98	0.02	0.02
	Medium off-training PA	0.36	0.66	0.42	0.34
	Higher off-training PA	0.16	0.38	0.56	0.62
	Lower training responses	0.48	0.98	0.02	0.02
	Medium training responses	0.36	0.66	0.42	0.34
	Higher training responses	0.16	0.38	0.56	0.62
Medium sleep	Lower off-training PA	0.81	1.00	0.00	0.00
	Medium off-training PA	0.10	0.37	0.47	0.63
	Higher off-training PA	0.09	0.31	0.53	0.69
	Lower training responses	0.41	0.72	0.44	0.28
	Medium training responses	0.46	0.95	0.07	0.05
	Higher training responses	0.13	0.42	0.49	0.58
Higher sleep	Lower off-training PA	0.44	0.99	0.00	0.00
	Medium off-training PA	0.41	0.96	0.26	0.04
	Higher off-training PA	0.15	0.77	0.74	0.23
	Lower training responses	0.14	0.77	0.71	0.23
	Medium training responses	0.56	0.99	0.00	0.00
	Higher training responses	0.30	0.95	0.29	0.05

*PA* physical activity.

For the Medium sleep group results showed the first dimension was characterized by a Lower off-training PA profile (as the relative inertia and cosine^2^ values exhibit) (see Table 2 and Fig. 2b) and a second dimension was most related to the Higher and Medium PA profiles. Regarding the training response, a similar trend was observed for Medium and Lower responses (dimension 1, 72.97% of inertia) in contrast with the Higher responses (dimension 2, 27.03% of inertia). Analyzing the quadrants in Fig. 2b, correspondence can be observed between Higher off-training PA and Higher training responses, Medium off-training PA and Lower training responses, and possibly independence from the Lower off-training PA profile and Medium training responses.

For the Higher sleep group results showed the first dimension was characterized by the Lower and Medium off-training PA profiles (see Table 2 and Fig. 2c) and a second dimension was most related to the Higher off-training PA. For the training responses, dimension 1 (94.43% of inertia) was described by Medium and Higher responses, having dimension 2 (5.72% of inertia) a relation with the Lower responses. The quadrants from Fig. 2c are less clear. It is possible to perceive a slight correspondence between Medium off-training PA and Medium training responses and independence from the additional off-training profiles and training responses.

## Discussion

This study quantified and assessed the relationship of young soccer players' off-training PA and training responses on sleep quality. Results provided valuable insight information into the interactive nature of lifestyle and performance by identifying correspondences between these dimensions.

A major outcome of this study was that young soccer players revealed alarming SB patterns, with all clusters demonstrating long-periods of SB during off-training periods. It is likely players considered time spent in soccer training is enough to minimize the harmful effects of sedentariness, therefore neglecting this issue. However, this mindset is flawed as recent evidence has found SB and body fat levels to be independent of the amount of MVPA^28^. Moreover, high physical inactivity can reduce metabolic improvements deriving from exercise^29^. As players were only monitored on weekdays, it is plausible that the PA profiles were shaped by school time and activities. Therefore, schools should assume an important role in changing SB, especially with evidence suggesting that brief periods of PA during school also promotes academic improvements^30^. Medium and Higher PA groups achieved the World Health Organization (WHO) daily recommended levels of MVPA (i.e., considering the mean wear time for the sample of this study was of ten hours), whereas the Lower PA group did not^31^. This evidence demonstrates the importance of organized sports to fulfill the daily MVPA recommendations. But also highlights a concern for those unable to reach the required minimum physical activity levels, as this lack of activity is often carried into adulthood^10^, with the additional likelihood of reduced sports participation.

It is widely known that the training stimulus and resulting training responses are influenced by the environmental constraints imposed on soccer players^32^. Accordingly, the cluster Medium training responses suggests the use of training sessions characterized by the presence of technical analytical drills or small-sided games, normally conducted with a small number of players and performed in small spaces. This elicits an increased amount of changes of direction and physical contacts between players, consequently increasing the number of impacts^14^. Conversely, training drills to improve and enhance ball circulation tactics, elicit higher distances covered and more high-intensity running and sprinting, which may explain the cluster Lower training profiles^15^. The training responses of cluster Higher illustrated the highest training workload, possibly due to training sessions including game situations, performed in a larger area-to-player ratio, leading to greater distances covered at higher speeds, and an increased number of impacts^14,33^. It is well documented that even in youth soccer, coaches frequently mimic the physical and tactical competition demands in training^15^. Therefore, it is hypothesized that training responses may be more influenced by the coaches training plan as opposed to individual player abilities (i.e., technical, tactical, motivation).

To recover from the stress imposed on players by the training stimulus, sleep is considered the primary recovery mechanism^24,34^. Although literature suggests getting enough high-quality sleep is critical for young athletes^20,26^, clusters Lower and Medium sleep presented a sleep efficiency below 80%. Consequently, these young soccer players' are experiencing sub-optimal sleep, which may have may be detrimental for sporting and academic performance^20,22^. Whereas, cluster Higher sleep presented satisfying values in all sleep quality variables^23,35^. The observed differences highlight the need to account for young players’ sleep and to assess the efficacy of simple, practical and non-pharmacological sleep hygiene strategies, that can be self-administered before bedtime, to improve sleep-related variables and sleep quality^25^.

Although there is an emerging awareness about the relationship between daytime SB, PA, training demands and sleep quality, most studies only focus on the effects of training events on sleep^21,23,35,36^. However, the amount of time spent in soccer training represents a reduced part of a full day (i.e., 1–2 h), while off-training PA and SB includes most of the day, whereby both dimensions should be taken into consideration when analyzing sleep. Therefore, this study is the first to report the correspondence between off-training PA and training responses, on sleep quality.

Interestingly, when sleep quality is Lower, the Medium and Higher off-training PA profiles corresponded to Higher and Medium training responses. Although speculative, these results may indicate that high PA levels during the day, considering off-training and training PA concomitantly, may be responsible for Lower sleep quality nights. Furthermore, an increased volume of impacts demonstrated through Higher and Medium training responses could cause significant fatigue and muscle soreness^23^, which combined with the off-training PA profiles that meet or exceed the daily recommended guidelines, may further contribute to impaired sleep.

Slightly different correspondences were observed when sleep quality was Medium. Similarly, to the Lower sleep correspondences, high PA levels during the day, illustrated by the correspondence between Higher off-training PA and Higher training responses potentially acted as a bias to sleep quality, considering the low sleep efficiency and high sleep disruption observed. However, this group showed an intriguingly lower sleep latency among sleep quality clusters. These somewhat contradictory results might derive from other circumstances unaccounted for. For example, sleep may be affected by post-training shower temperature, and food consumption or use of light-emitting technology devices before sleep^24,25^. The Medium sleep group showed a correspondence between Medium off-training PA and Lower training responses. Considering that Lower training responses gathered lower training impacts, compared to other groups, may suggest these different correspondences reveal certain workload metrics that might be detrimental to young players' sleep quality. Indeed, training workload volume and intensity might dictate the young players’ wellbeing status^23^, which combined with the off-training PA throughout the day can affect their sleep quality.

Contrastingly, the absence of correspondence between off-training and training response profiles leads to Higher sleep quality. While the remaining sleep clusters expose a clear association between the off-training profiles with longer MVPA and training responses, the Higher sleep group isolates the Higher training response and the Higher off-training PA profile. But there was an almost non-existent correspondence between the Medium off-training PA profile and the Medium training responses. These findings combined with previous results suggest the existence of an ideal interaction between lifestyle and performance. Therefore, providing relevant knowledge to athletes’ development programs, highlighting the need of monitoring talented players’ PA, directly and indirectly, related to the training. This will help create a comprehensive overview of wellbeing that may be neglected by a single analytic analysis.

Additionally, regardless of the sleep group, Fig. 2 portrays the Higher and Medium off-training PA profiles on the left quadrants, and the Lower off-training PA profile on the right quadrants, emphasizing that much of the total inertia is due to the off-training sedentary PA and MVPA. Moreover, none sleep clusters exposed a correspondence between the Lower off-training PA group and any training responses. Possibly, the determinants contributing to this lack of correspondence are related to additional factors, such as the SB type, the complex nature of team sports training and sleep hygiene practices^9,25,35,36^. Conversely, the Medium off-training PA profile corresponds with all the training responses, despite exhibiting a slight association to Medium training responses in the Higher sleep group. Thus, it is suggested that players' training responses are more reliant on the prescribed training load by the coach than players' pre-training PA.

This study adds relevant information regarding young soccer players' sleep quality and its relationship with off-training PA and training responses, but the interpretation of these results should be taken cautiously, considering only eleven players from a single team were involved. Concomitantly, assessing players' wellbeing (e.g., muscular soreness) or subjective perceived exertion after soccer training would have allowed describing the training demands in more detail. Future research should focus on how sleep quality and prolonged inadequate sleep habits are related to off-training PA, training performance, accumulated fatigue and muscle soreness of subsequent days. Furthermore, further research is recommended to examine whether manipulating off-training and training PA may be beneficial or detrimental for young players health and long-term performance development.

## Conclusion

This study presents insightful information about young soccer players off-training PA, training responses and sleep quality, providing an improved understanding of the interactive nature of their lifestyles. The off-training PA profiles exposed distinct PA patterns, with high levels of SB, suggesting the importance of organized sports to achieve daily PA recommendations. The differences in training responses highlighted that players training performance depends considerably on the training stimulus planned by coaches. Sleep clusters exposed that young soccer players often have a disrupted sleep pattern, suggesting the importance of health and sports organizations in supporting the implementation of sleep hygiene policies to improve sleep quality. Most importantly, the results showed that Higher and Medium training responses combined with Medium and Higher off-training PA led to Lower sleep quality. Accordingly, training workload volume and intensity may undesirably affect young players wellbeing status, which combined with the nature of the off-training PA may impair sleep quality. Conversely, no evident correspondence was found between off-training behavior and training responses, concerning Higher sleep quality. In conclusion, there is a need to account for and optimize young players’ training and off-training time, to improve their sleep quality. Therefore, youth sports organizations should promote a holistic approach to soccer players development, targeting an appropriate balance between lifestyle, performance and health.

## Methods

### Participants

Sixteen, regional level under-15 male outfield soccer players (age, 13 ± 0.58 years old; body mass, 50 ± 9.22 kg; height, 160 ± 9.64 cm), volunteered to participate in this study. Criteria for players inclusion were to partake in three training sessions (with, at least, 90 min’ duration) and one competitive match per week. Players, their legal guardians, and their coach were fully informed about the procedures, purpose and risks of the study and provided written informed consent before the study commenced. No players reported any musculoskeletal, neurological, or orthopedic injury that may impair their participation. The study protocol was performed per the recommendations of the Declaration of Helsinki^37^ and was approved in compliance with the guidelines stated by the Ethics Committee of the University of Trás-os-Montes and Alto Douro, based at Vila Real, Portugal (PCE43/2019).

### Design

**Figure 3:**
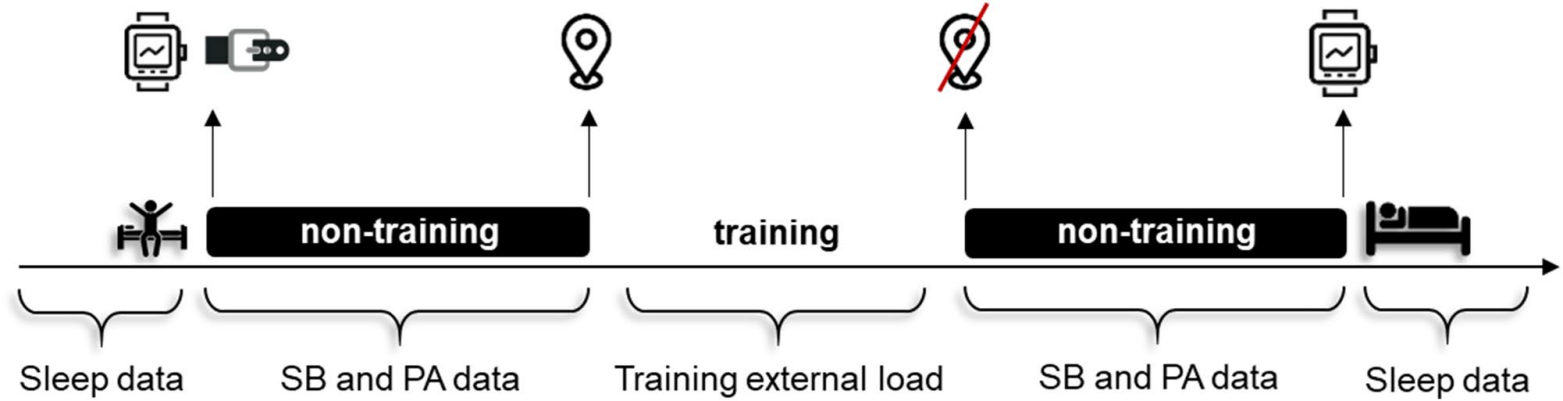
Representation of a data collection day.  = wake up;  = go to the bed at night;  = wear GPS;  = remove GPS;  = accelerometer on the elastic belt;  = accelerometer worn on the wrist. *PA* physical activity, *SB* sedentary behavior.

Off-training PA, sleep, and soccer training data for all players were collected over four weeks (during weekdays), throughout the in-season period. Players were instructed to wear the accelerometer on soccer training days (i.e., Monday, Wednesday and Thursday), from getting out of bed in the morning, until they woke up the next morning (except during water-based activities, personal hygiene care and soccer training sessions). Throughout the waking hours, the accelerometer was firmly placed and adjusted on the players’ waist via an elastic belt over the right hip^6^. Whereas, overnight, players have worn the accelerometer on their non-dominant wrist (see Fig. 3). Players also completed a diary to record any removal of the device during the day, to report bedtime and time they got out of bed after the final awakening. It should be noted, that previously to the study start, the players and their legal guardians were given written and verbal instructions about how to wear the accelerometer properly and complete the diary. Furthermore, the day that the accelerometer was removed, the research team reviewed each diary with the participant to clarify unclear entries.

Regarding the soccer training sessions (i.e., from 6:30 p.m. until 8 p.m.), data was collected without any intervention of the research team regarding training volume and intensity. The training sessions included a warm-up; soccer drills that focused on technical and tactical skills acquisition and improvement; and soccer small-sided games. Players who failed to meet the inclusion criteria of at least three days of soccer training or were unable to provide sufficient accelerometer wear-time data of ten hours during the waking time and accelerometer worn overnight were excluded from the study. This led to a final sample of eleven players (age, 13 ± 0.5 years old; body mass, 51 ± 9.49 kg; height, 161 ± 9.05 cm) and 66 individual records.

### Data collection

#### Physical activity and sleep monitoring

ActiGraph GT9X-link accelerometer (ActiGraph, Pensacola, FL, USA) was used to obtain an objective assessment of players daily PA and sleep. The monitor was initialized at a sampling frequency of 30 Hz and data were processed using Actilife software V.6.13.4 (ActiGraph, Pensacola, Florida, USA). Sixty-second epochs were used in processing the accelerometer data^16^. Non-wear time was defined as periods of sixty or more consecutive minutes of zero counts and was excluded from analysis^6^. The cut points used to calculate the variables associated with PA intensity were adapted from prior research^38^ and standardized into the following levels: sedentary PA (0–180 counts/min); light PA (181–756 counts/min); moderate PA (757–1111 counts/min); vigorous PA (≥ 1112 counts/min); and MVPA (moderate PA + vigorous PA). It is noted that all PA variables were normalized according to the daily hours of accelerometer use. Players’ sleep quality was evaluated overnight (i.e., based on the information reported in the diary). The validity and reliability of wrist actigraphy were previously reported^39^. The Sadeh algorithm, valid for younger adolescents was used to assess sleep^40,41^. The variables considered in the analysis were onset latency (minutes), efficiency (AU) and SFI (AU). Sleep onset latency indicates the amount of time between bedtime and sleep onset time. Sleep efficiency specifies the percentage of time in bed spent asleep. The SFI is an index of restlessness during sleep expressed as a percentage, with higher levels of fragmentation indicating more disrupted sleep.

#### Training workload monitoring

Training workload was collected using individual Viper GPS units (Viper, STATSports, Newry, Ireland). The STATSports Viper system was previously reported valid and reliable, and their operation and handling are documented elsewhere^42^. After each training session, training workload data was downloaded using the respective software package (Viper PSA software, STATSports, Newry, Ireland) and exported for analysis^42^. The following variables were selected for analysis: total distance covered (m), distance covered at high-intensity running (13–18 km/h), sprinting (≥ 18 km/h), accelerations (0.5–3.0 m/s^2^), decelerations (− 0.5 to − 3.0 m/s^2^) and impacts (> 3 G’s forces)^43,44^. It is noted that all variables were normalized according to the time players spent on the pitch during each training session to understand session intensity.

### Statistical analysis

Firstly, a Shapiro–Wilk test assessed all data sets for outliers and normality. Then, a K-means clustering analysis was carried out to classify the players into different groups, in each of the following dimensions (variables used in the analysis): off-training PA (sedentary PA, light PA, moderate PA, vigorous PA and MVPA), training responses (total distance covered, high-intensity running, sprinting, accelerations, decelerations and impacts), and sleep quality (latency, efficiency and SFI). The algorithm was based on Euclidean distances and a maximum of three clusters for each dimension was specified. ANOVA and Tukey honestly significant difference (HSD) tests were also performed to compare the predictive influence of variables on the cluster groups obtained and to examine the groups mean differences according to the variables analyzed. Afterwards, the clusters from off-training PA and training responses were compiled in two-dimensional plots using a correspondence analysis. This method of analysis is a descriptive/exploratory technique designed to analyze simple two-way and multi-way tables containing some measure of correspondence between the rows and columns^45^. This technique provides information similar to those produced by a principal components analysis where the common position of categories in distance from the center of the presentation demonstrates the correlation or correspondence of the categories^46^. Three different plots were arranged to represent the results according to the sleep groups. All analyses were performed using StatSoft STATISTICA version 13.3 (StatSoft Inc., Tulsa, OK, USA).

## Data Availability

The datasets generated during and/or analyzed during the current study are available from the corresponding author on reasonable request.
